# Propafenone Poisoning of a Female Adolescent After a Suicide Attempt

**DOI:** 10.7759/cureus.16576

**Published:** 2021-07-23

**Authors:** Stergiani Keramari, Alexandros Poutoglidis, Frideriki Poutoglidou, Georgia Kaiafa, Michael Keramaris

**Affiliations:** 1 Second Department of Paediatrics, AHEPA University Hospital, Faculty of Medicine, School of Health Sciences, Aristotle University of Thessaloniki, Thessaloniki, GRC; 2 Otorhinolaryngology - Head and Neck Surgery, “G. Papanikolaou” General Hospital, Thessaloniki, GRC; 3 Clinical Pharmacology, Faculty of Medicine, School of Health Sciences, Aristotle University of Thessaloniki, Thessaloniki, GRC; 4 First Propaedeutic Department of Internal Medicine, AHEPA University Hospital, Faculty of Medicine, School of Health Sciences, Aristotle University of Thessaloniki, Thessaloniki, GRC; 5 Cardiology, General Hospital of Kastoria, Kastoria, GRC

**Keywords:** seizure, antiarrhythmic agent, propafenone, poisoning, arrhythmia

## Abstract

Propafenone is an antiarrhythmic agent for the management of ventricular and supraventricular tachycardia and atrial fibrillation. Propafenone poisoning is rare but may be life-threatening due to drug-induced arrhythmias. Electrocardiographic changes in PR, QRS, and QT intervals have been recorded. We present a case of a 15-year-old female adolescent who developed arrhythmias and convulsions due to propafenone intoxication, in an attempt to commit suicide. The outcome of the case was a full recovery from the arrhythmias and the seizures. The aim of this article is to highlight the possibility of a lethal intoxication by a common antiarrhythmic drug. Our case aims to present our therapeutic strategy that relies mainly on close monitoring of patients and cardiac output support.

## Introduction

Propafenone is an antiarrhythmic IC class medication [1-4]. The main indication of the use of propafenone is the prevention of ventricular and supraventricular tachycardia and atrial fibrillation [5]. Patients with structural heart disease are not eligible for treatment with propafenone due to the high risk of developing proarrhythmic events [6].

Overall, propafenone inhibits the action potentials on the cardiac sodium channels. It also inhibits the β-adrenergic receptors, and the calcium receptors and has a negative inotropic action. Side effects of propafenone include life-threatening electrocardiogram (ECG) anomalies, such as ventricular arrhythmias, ventricular fibrillation or tachycardia, asystole, Torsades de Pointes, and prolongation of the QT or QRS interval [7-8]. Unfortunately, there is currently no known antidote for propafenone intoxication [9].

Propafenone intoxication remains a clinical challenge, even for the most experienced cardiologists. Cases are sparse in literature and a wide range of guidelines have been proposed. Our case report highlights the efficacy of inotropic agents for cardiac function support after propafenone intoxication.

## Case presentation

A 15-year-old female adolescent was admitted unconscious to our emergency department (ED). On her arrival, generalized tonic-clonic seizures were recorded and a loss of bladder control was observed. The patient's parents reported no history of health issues or substance abuse. Family history was also unremarkable. Intravenous diazepam was administrated at a dose of 1 mg/kg and a resolution of the convulsions was achieved. Nevertheless, consciousness was not regained.

**Figure 1:**
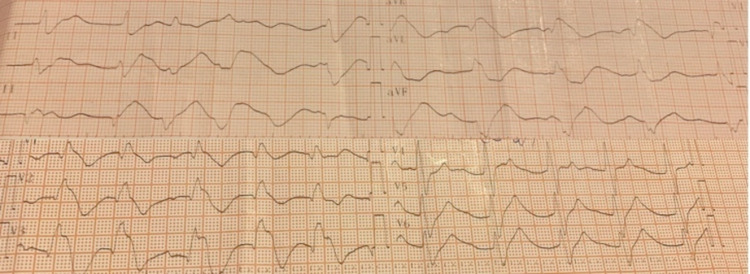
The initial ECG. PR interval prolongation, QT prolongation, and QRS prolongation to 170 ms and a corrected QT interval (QTc) of 490 ms ECG, electrocardiogram

Physical examination revealed a Glasgow Coma Scale (GCS) of 9/15, a bradycardia (48 bpm), a low blood pressure (60/45 mmHg), a respiratory rate of 10 breaths/min, and a temperature of 35.6°C. Blood glucose level was within the normal range (finger stick 109 mg/dL). The initial ECG showed a PR prolongation, a QT prolongation, a QRS prolongation to 170 ms, and a corrected QT interval (QTc) of 490 ms (Figure 1). The initial arterial blood gases (ABGs) are shown in Table 1. The initial laboratory work-up included a complete blood count (CBC), blood urea nitrogen, serum creatinine levels, and an electrolyte panel, which were all normal. Her chest radiograph was also normal. 

**Table 1:** ABGs at different time intervals. BE, base excess; ABGs, arterial blood gases

ABGs			
	On admission	After inotropic agents administration	Five hours later (full recovery)
pH	7.062	7.3	7.42
PCO_2 _(mmHg)	46.2	33.92	33.7
PO_2 _(mmHg)	69.2	90.62	82.9
HCO_3 _(mmol/L)	13.2	16.8	22.1
ΒΕ (mmol/L)	-17.3	-9.8	-2.7

**Figure 2:**
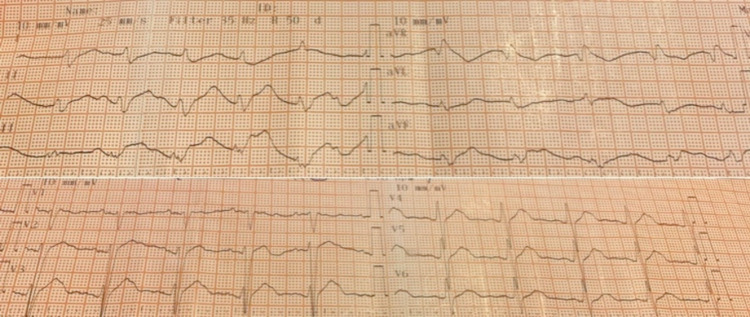
ECG after inotropic agent administration. PR interval prolongation, QT prolongation, and QRS prolongation to 160 ms and a corrected QT interval (QTc) of 478 ms ECG, electrocardiogram

**Figure 3:**
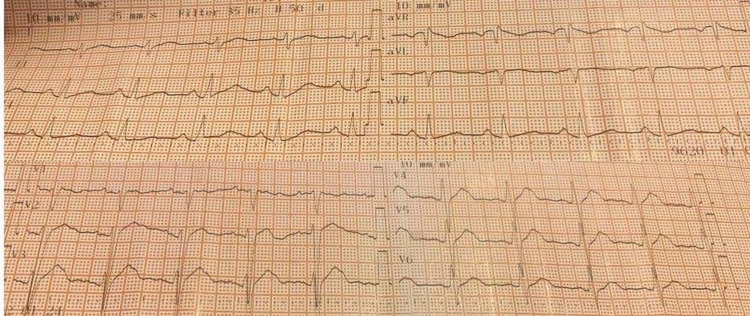
ECG after recovery. PR interval 180 ms, QT and QRS 110 ms, and a corrected QT interval (QTc) of 420 ms ECG, electrocardiogram

Oxygen was supplied via a simple face mask at a flow rate of 8-10 L/min. On the suspicion of opioid or benzodiazepine overdose, naloxone (0.6 mg) and flumazenil (0.62 mg) were administrated intravenously. Two minutes later, the patient regained consciousness but the bradycardia, the hypotension, and the ECG morphology were not improved significantly. The patient reported the consumption of 10 propafenone tablets (225 mg per tablet), which are equivalent to 2250 mg, five hours before. Inotropic agents, dopamine (4 μg/kg/min) and dobutamine (4 μg/kg/min), were administered under close monitoring. The doses were increased at 6 μg/kg/min of dopamine and 6 μg/kg/min of dobutamine. The new ABGs are presented in Table 1. One hour after the inotropic agents’ administration, an improvement in diuresis was observed. The heart rate increased (65 bpm) and the ECG findings were improved (Figure 2). Five hours later, full recovery was observed. The blood pressure was at 78/59 mmHg, the heart rate was 80 bpm, and the ECG findings (Figure 3) and the ABGs values (Table 1) returned to normal. The patient was discharged 24 h after admission in excellent condition and was referred to psychiatric consultation. 

## Discussion

Propafenone is a commonly used antiarrhythmic IC class drug used for the treatment and control of ventricular and supraventricular tachycardia and atrial fibrillation. There are limited data regarding propafenone intoxication which could be explained by the rarity of these cases. Propafenone's mechanism of action mainly relies on the stabilization of the voltage in the myocardial cell membranes by blocking the sodium channels, the β-adrenergic receptors, and the calcium channels. Propafenone is also characterized by a negative inotropic action [10-11]. The maximum concentration in serum after oral administration is achieved within three to six hours. Following intoxication, the most life-threatening ECG abnormalities are expected to occur within this time interval.

There are two genetically determined patterns of propafenone metabolism. Patients can be categorized either as extensive metabolizers (EMs) or as poor metabolizers (PMs). EMs account for 90% of the patients. Propafenone is metabolized in 5-hydroxypropafenone from the cytochrome P-450 CYP2D6 isoenzyme and in N-depropylpropafenone from the CYP3A4 and CYP1A2 isoenzymes. Propafenone has a short elimination half-life (5.5 ± 2.1 h). PMs, who account for the 10% of patients, exhibit a decreased action of the isoenzyme CYP2D6 and in those patients, a longer half-life time of propafenone (17.2 ± 8 h) has been reported [5, 12-14]. Thus, close monitoring over a 24-h period is required. Previous studies have shown that the β-blockade effect appears in both phenotypes in a propafenone intoxication; however, the PMs may have a higher propafenone concentration and a greater β-blockade effect [15].

There are several rare adverse effects associated with propafenone, such as allergic reactions, gastrointestinal manifestations, the elevation of liver enzymes, white blood cells disorders (agranulocytosis), headaches, peripheral neuropathy, orthostatic hypotension, ventricular fibrillation, heart failure, and bradycardia. Toxicity of propafenone may lead to coma, cardiac arrest, and arrhythmias (sinus arrest, atrial fibrillation, ventricular tachycardia, prolongation of the PR, QRS, and QT interval widening, Brugada phenocopy, and ventricular fibrillation) [5-7, 14-16]. Propafenone overdose may be fatal [1-4]. Inotropic agents, IV hypertonic bicarbonate, and IV lipid emulsion have been used for the management of propafenone intoxication. Unfortunately, currently, there is neither an antidote nor a specific treatment [17-19]. In our case, tonic-clonic seizures were observed, as a severe neurological manifestation of propafenone intoxication [18]. Seizures are a common sign of propafenone intoxication; however, the reason for the convulsion is unknown. It is unclear whether the seizures are a side effect of the propafenone per se in the central nervous system or it is due to the cerebral hypoperfusion because of the low cardiac output. 

## Conclusions

This case report highlights the severity of intoxication by propafenone which can lead to bradycardia, cardiac failure, seizures, loss of consciousness, and potentially death. Immediate treatment, close monitoring and support of the cardiac output are critical for full recovery. Our case report demonstrates that inotropic drugs should be considered as an effective treatment option for the management of propafenone intoxication.
